# SCELLECTOR: ranking amplification bias in single cells using shallow sequencing

**DOI:** 10.1186/s12859-020-03858-y

**Published:** 2020-11-12

**Authors:** Vivekananda Sarangi, Alexandre Jourdon, Taejeong Bae, Arijit Panda, Flora Vaccarino, Alexej Abyzov

**Affiliations:** 1 Department of Health Sciences Research, Center for Individualized Medicine, Mayo Clinic, Rochester, MN 55905 USA; 2 Child Study Center, Yale University, New Haven, CT 06520 USA; 3 Department of Neuroscience, Yale University, New Haven, CT 06520 USA

**Keywords:** MDA, Single cell, Whole genome amplification

## Abstract

**Background:**

The study of mosaic mutation is important since it has been linked to cancer and various disorders. Single cell sequencing has become a powerful tool to study the genome of individual cells for the detection of mosaic mutations. The amount of DNA in a single cell needs to be amplified before sequencing and multiple displacement amplification (MDA) is widely used owing to its low error rate and long fragment length of amplified DNA. However, the phi29 polymerase used in MDA is sensitive to template fragmentation and presence of sites with DNA damage that can lead to biases such as allelic imbalance, uneven coverage and over representation of C to T mutations. It is therefore important to select cells with uniform amplification to decrease false positives and increase sensitivity for mosaic mutation detection.

**Results:**

We propose a method, Scellector (single cell selector), which uses haplotype information to detect amplification quality in shallow coverage sequencing data. We tested Scellector on single human neuronal cells, obtained in vitro and amplified by MDA. Qualities were estimated from shallow sequencing with coverage as low as 0.3× per cell and then confirmed using 30× deep coverage sequencing. The high concordance between shallow and high coverage data validated the method.

**Conclusion:**

Scellector can potentially be used to rank amplifications obtained from single cell platforms relying on a MDA-like amplification step, such as Chromium Single Cell profiling solution.

## Background

Somatic mutations acquired in each cell during and after embryogenesis are passed to the descendant cells such that, within the same individual, different populations of somatic cells have slightly different DNA, resulting in genomic mosaicism. The accumulation of somatic mutations increases with age [1–3], and is also affected by environmental factors like tobacco smoking and alcohol consumption [4]. Somatic mutations can not only cause cancer but also diverse neurological diseases, including cortical malformations, epilepsy, intellectual disability, and neurodegeneration [5, 6]. Some somatic mutations might give the cells proliferative advantage, and ultimately cause cancer, or can affect the cellular functions without a proliferative effect. This makes the detection of mosaic mutation important for understanding the mechanism of various diseases.

Although whole genome sequencing of bulk tissue has been used for detecting somatic mutations, it is not sensitive enough to detect mosaic mutations present below 1% variant allele frequency (VAF), i.e., a heterozygous mutation present in less than 2% of the cells. This hurdle has been overcome by single-cell DNA sequencing (scDNA-seq) which in recent times has emerged as an efficient tool for studying mosaic mutations [7–9]. Since the starting DNA amount in a single cell is very low, an additional step of DNA amplification is required. There are two types of broad methods for DNA amplification: cell cloning and enzymatic Whole Genome Amplification (WGA). Depending on the experimental design one of the two methods can be used. WGA methods, unlike cell cloning, directly isolates extracted DNA from single cells and then amplify it, making it possible to sequence the DNA of cells which cannot be cultured, such as neurons. There are three types of WGA methods: DOP–PCR (Degenerate Oligonucleotide–Primed Polymerase Chain Reaction) [10], MDA (Multiple Displacement Amplification) [11] and MALBAC (Multiple Annealing and Looping–Based Amplification Cycles) [12], each having its advantages and drawbacks. MDA is the most widely used method for WGA owing to its longer fragment length (up to 70 kbps), low error rate during amplification and higher fraction of the genome being amplified as compared to the other WGA methods [13].

MDA is an exponential amplification method where the DNA is amplified using a high fidelity phi29 polymerase with proofreading activity under isothermal conditions [11]. However, phi29 polymerase is sensitive to template fragmentation happening during cell lysis as well as presence of blocking sites where DNA damage prevents amplification. This may lead to uneven coverage, over-fragmented or completely damaged DNA, which may further lead to allelic imbalance when one of the alleles is under-amplified and the other allele is over-amplified. Even though MDA results in high yield of DNA material, introduction of biases such as allelic imbalance and over representation of C to T mutation introduced during lysis can affect the variant detection downstream.

Before moving forward with high coverage Whole Genome Sequencing (WGS), it is important to select cells with successful amplification, exhibiting little or no biases. Uneven amplification, with the ultimate manifestation of allelic drop-outs (i.e., random and drastic overrepresenting of one allele over the other), challenges separating false positives from real somatic variants. For example, deamination of cytosine happening during cell lysis on one strand of one allele are expected to have 25% allele frequency in a balanced amplification and, based on that, can be marked as artifact. However, if the other non-deaminated allele is not amplified, the allele frequency for the artifact will become 50%, making it indistinguishable from a heterozygous variant. So, using a cell with high allele drop-out rate will result in more false positives and reduce sensitivity, as variants in dropped out regions cannot be discovered.

PCR can be used as a first quality control to test the presence of several random genomic loci, usually chosen on different chromosomes, in the amplified DNA. Multiplex-PCR of 4 loci in one PCR reaction can for instance be used as a rapid quality control where cells are considered to have good quality amplification if at least 3 loci are detected [14]. However, this test is quite limited as there might be regions outside of the 4 loci with un-uniform amplification. Similarly, failing the test doesn’t imply low amplification quality outside of the 4 loci. It is therefore essential to look at the genome as a whole. A few methods for checking amplification quality in silico from WGS data were proposed. Statistical models have been used to detect amplification bias using depth of sequence [15]. Amplification quality prior to sequencing has also been determined by using power spectral density to estimate uniformity of amplification which can be otherwise masked by non-unique read mapping, assembly gaps and locus dropouts (both alleles are not amplified) [16], and median absolute difference (MAPD) [17]. However, these methods either rely on at least 20×–30× coverage or do not evaluate allelic imbalance, which is important to access to have full coverage of all haplotypes in a cell.

Here, we describe a method to determine the extent of allelic imbalance introduced by MDA into the amplified DNA using shallow (< 1×) sequencing coverage. The method is based on considering allele frequency distribution of the heterozygous SNPs, which, for diploid genome, should have a Gaussian distribution centered around 50%. In case of a non-uniform amplification, the distribution of a majority of the SNPs will support homozygosity, suggesting high rate of allelic drop-outs during amplification.

## Results

**Fig. 1:**
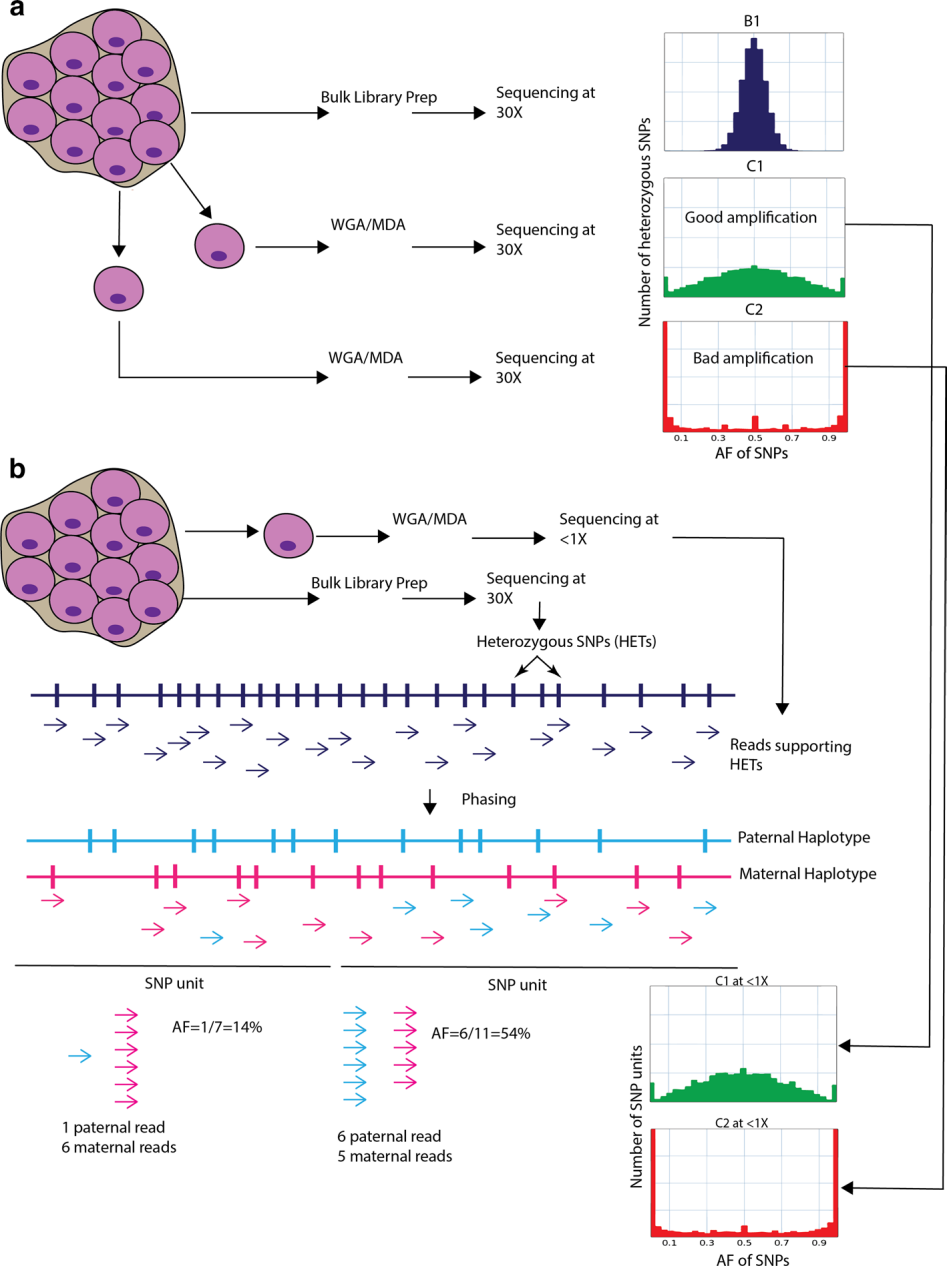
Concept and workflow of the approach. **a** VAF distribution of HETs at 30× sequencing coverage in three cases: Bulk sample, uniformly amplified cell, and un-uniformly amplified cell. The distribution from bulk shows a peak around 50%, which is expected. Then we have a single cell sequenced at 30× with good amplification. The allele frequency plot still has a peak around 50%, but not as sharp as the bulk sample. The last example is a single cell also sequenced at 30× but with non-uniform amplification. **b** Conceptual description of the approach. First, SNPs are phased. The reads supporting the SNPs are divided into two haplotypes, named maternal and paternal, although the exact origin of each haplotype is unknowns. With less than 1 read supporting each SNP (coverage < 1×), multiple SNPs are merged to form a SNP unit. Reads supporting SNPs within the SNP unit from only one haplotype are used to calculate the allele frequency over that SNP unit. The allele frequency plot for high coverage data closely resembles the one from shallow coverage data

Each single cell sequencing experiment can involve hundreds of single cells. After WGA, not all cells are amplified uniformly owing to the allelic imbalance described earlier. Allelic imbalance can be checked from the VAF of heterozygous SNPs (HETs) in the cell. When sequencing in bulk, the VAF distribution of HETs should be centered at 50% and be bell-shaped (Fig. 1a). For a balanced single cell amplification, the distribution should follow the same shape, but can have wide dispersion. For an unbalanced amplification the distribution will not be bell-shaped, and one allele will be drastically overrepresented over the other one.

At shallow coverage, most SNPs will either have just a few or no reads supporting them, making assessment of amplification quality impossible (Additional file 1: Fig. S1). Therefore, the underlying idea of the method is to judge the quality of amplification based on VAF of multiple consecutive HETs from the same haplotype, rather than on individuals HETs. This however requires that HETs are phased to haplotypes. When HETs from the same haplotype are combined, it allows reaching per unit read counts that are comparable to those for individuals HET at high sequencing coverage (Fig. 1b). Furthermore, it is important to note that the implicit assumption is that multiple consecutive SNPs are amplified together. For MDA, which is known to have around 50–70 kb amplified fragments [11], it is a valid assumption.

Our QC workflow proceeds as follows (Fig. 1b). First, we determine HETs from a bulk sample sequenced at high coverage. These SNPs are then phased into maternal and paternal haplotypes using the SHAPEIT2 method [18, 19], which has been shown to be the most accurate method for phasing sets of known genotypes [20]. Multiple consecutive HETS are merged to form a SNP unit. The number of SNPs in the SNP unit is determined by the coverage of the cell. For a high coverage data (~ 30×) with 100 bp reads, we use each heterozygous SNP for calculating VAF across the genome. Proportionally, for coverage of 0.3× with 100 bp reads, the number of SNPs to be used in a SNP unit is 100 (30× divided by 0.30×). The number of SNPs in a SNP unit is inversely proportional to the coverage. The reads supporting SNPs within the SNP unit from only one haplotype are used to calculate the allele frequency over that SNP unit. An allele frequency plot is then generated using all the SNP units similar to how it is done for VAF distribution of individual HETs at high coverage.

**Fig. 2:**
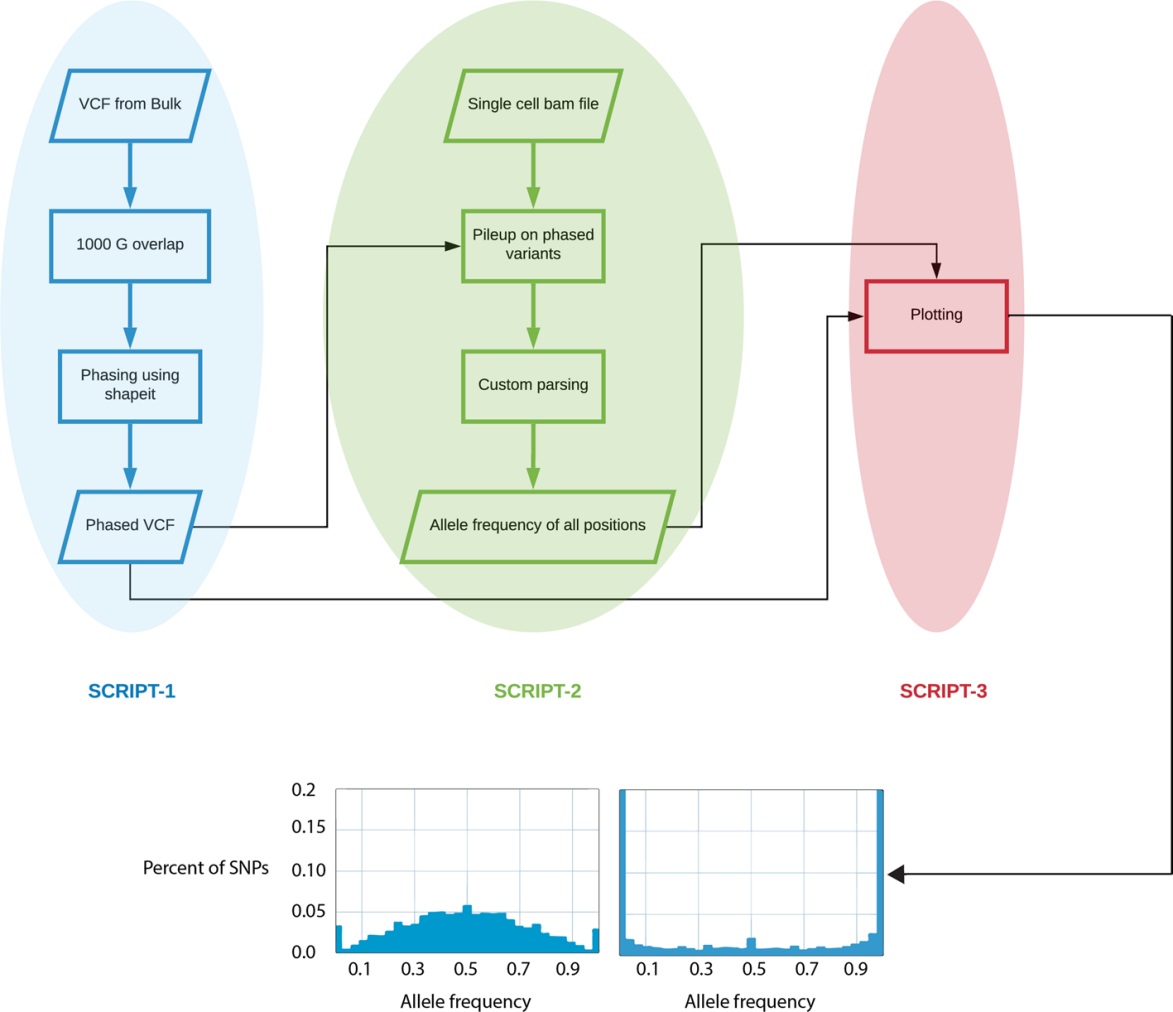
Flowchart of method implementation. Script 1 through 3 should be executed in sequence, however, they are independent of each other and as long as the input are correct, user can start with any script. The final Script-3 produces a VAF plot for each sample. Two examples of uniformly (on the left) and un-uniform (on the right) amplified cells are shown

The described approach was implemented in a modular pipeline written in python. The pipeline consists of three scripts and each script can be run independent of each other as long as the user has the required input file (Fig. 2). Script-1 takes a VCF file from the bulk sample (the germline SNPs can be called either using sequencing or any other genotyping methods), subsets it into SNPs present in the catalogues of germline variants provided by the 1000 Genomes Project [21], followed by phasing the SNPs using SHAPEIT2, and provides a phased VCF file. Script-2 uses the phased VCF and the low coverage bam file from the single cell to generate allele frequency over all SNPs. Script-2 can be used independently of Script-1, which allows users to use phasing tools other than SHAPEIT2 as long as the input is in VCF format. Script-3 takes the allele frequency of the SNPs from Script-2 and the phased VCF from Script-1 (or user specified phased VCF) to generate the allele frequency plot and ranks cells using only one of the parental haplotypes. The SNP unit is automatically calculated and applied by default using the equation mentioned earlier. It must be noted that, for coverage lower than 0.3×, the number of SNP in a SNP unit increases beyond a single MDA amplified fragment and can lead to averaging of multiple amplified fragments. In case of very low coverage, this may lead to a poorly amplified cell being represented as a good cell (Additional file 1: Fig. S2). For this reason, we also provide an option where the user can override this with their own SNP unit. The result of the final script is a plot showing the distribution of the SNP units allele frequency (Fig. 2).

To test our method, we did shallow sequencing on human iPSC-derived single neuronal cell which were amplified using MDA. Cellular DNA was sequenced at various read coverages (0.11–0.38) and data were then processed through Scelector. SNP unit size was determined for each cell based on read coverage. Based on the obtained VAF distribution and allelic dropout rate, we ranked single cells as having good, moderate and bad amplifications. Cells with standard deviation less than 0.26 were considered as uniformly amplified (good) cells and cells with standard deviation between 0.26 and 0.35 were considered moderate cells (Additional file 1: Fig. S3). Bad cell with standard deviation higher than 0.35 were used as negative control. Out of 14 single cells with shallow sequencing we picked 2 good cells, 5 moderately good cells, 1 bad cell as a negative control and 1 cell (i.e., B01) for which amplification quality could not be determined due to too shallow (0.11×). The selected 8 cells were then re-sequenced at high coverage (at least 30×) using DNBseq platform and their amplification quality was assessed through VAF distribution for individual HETs.

**Fig. 3:**
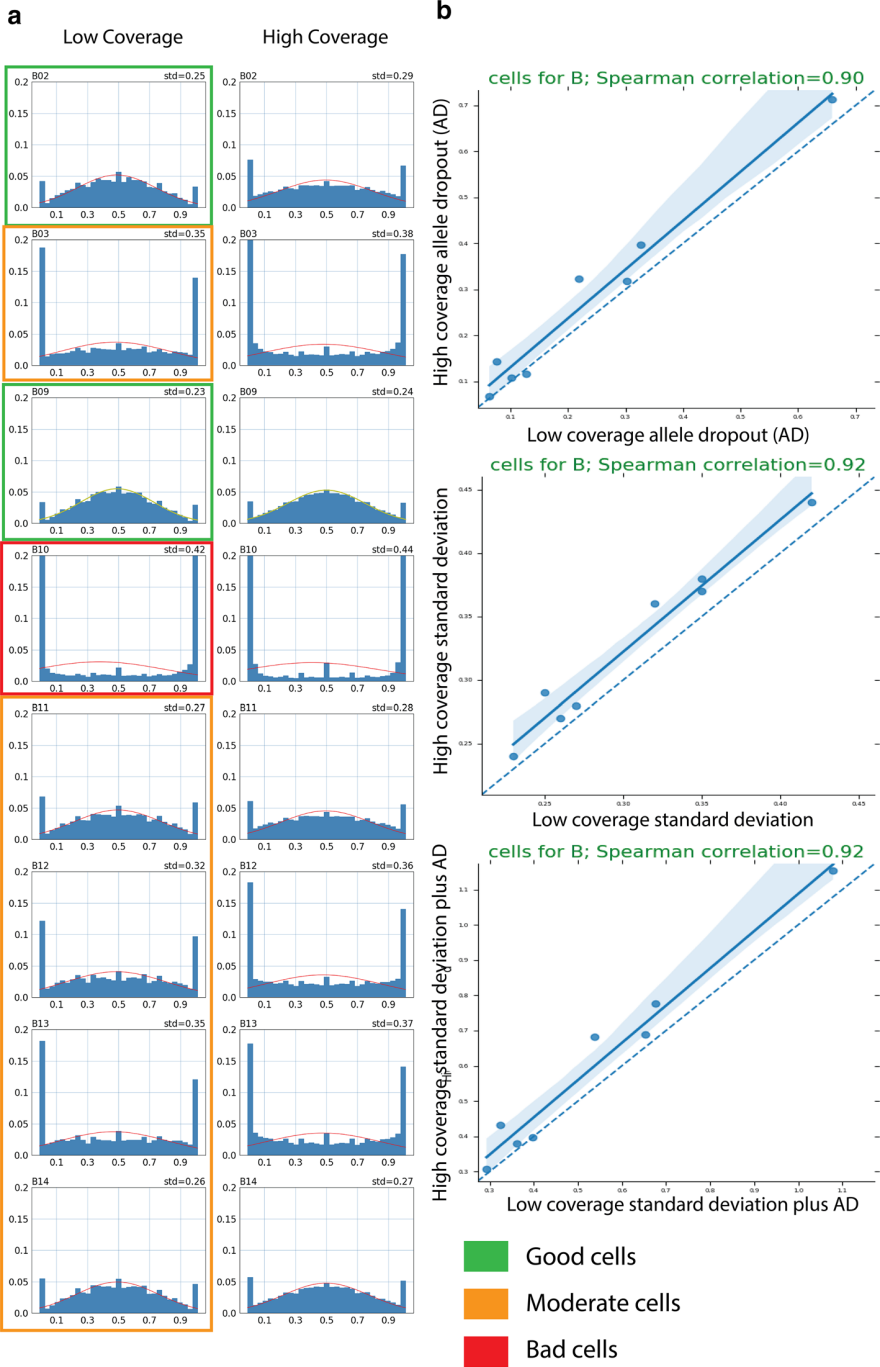
Validation of Scellector using 9 cells subjected to shallow sequencing by high coverage sequencing. **a** Side by side comparison of the allele frequency plots from shallow coverage and high coverage. **b** Scatter plot showing high correlation between the shallow and high coverage. Three comparison using allele dropout, standard deviation and a combination of standard deviation and allele dropout (AD) show similar results

We saw a good concordance between shallow and deep coverage indicating that our method can accurately estimate the effects of non-uniform amplification from shallow sequencing data (Fig. 3a). We noticed that the standard deviation was slightly higher in the deep coverage data. We reasoned that this is because SNP units can span more than one MDA amplified fragments (of typical size of 50–70 kbp), which averages the amplification bias making it seem less to that of high coverage data. Using Spearman correlation, we estimated the concordance between high and low coverage data for the same cells to be 0.92 (Fig. 3b). We also found similar high correlations using allelic dropout rate only and additive effects of standard deviation and allelic drop out. Above mentioned cell B01, which was excluded due to low coverage also turned out to be well amplified (Additional file 1: Fig. S4).

### Usage guidelines

Bias in amplification may result not only in allelic imbalance but also in non-uniform coverage across genome. We found that the quality of amplification measured using our method correlates with coverage uniformity (Additional file 1: Fig. S5) and more balanced amplification likely to results in more reliable CNV calls (Additional file 1: Fig. S6). Furthermore, there is an increase in percent of not covered bases as the standard deviation and allelic dropout rate increases (Additional file 1: Fig. S5). Additionally, our analysis suggests that our method is more sensitive than pairwise bin comparison approach like MAPD (Additional file 1: Fig. S10). Finally, allelic imbalance is independent of combination of nucleotide substitution in SNPs (Additional file 1: Fig. S7). Therefore, we suggest haplotype imbalance as a universal indicator of biased amplification.

Currently, VAF distribution of HETs from bulk is the target that none of single cell amplification methods can achieve. We also note that there exists no clear standard about what is good and what is bad amplification. To address this issue, we take an empirical approach by considering amplification quality of single cell from different independent studies, including our own, Lodato et al. [7] and Sanchez-Luque et al. [22] data. From these studies the consensus emerges that standard deviation of ~ 0.27 with allelic dropouts of less than 10–15% indicate the best currently achievable amplification (Additional file 1: Fig. S8). As discussed above, using SNP units large than typical length of amplified fragments leads to averaging amplification bias and we therefore recommend using for QC coverage of ~ 0.3× of higher. Using these guidelines, we estimated that study of single cell genomes can save a significant amount of funds on sequencing (Additional file 1: Fig. S9).

## Discussion

Single cell omics experiments are becoming increasingly crucial for mapping cell heterogeneity in tissues and organs from many different perspectives, from transcriptomics and DNA variations to epigenomic such as chromatin accessibility (i.e. scATAC-seq). Single cell sequencing experiments can be very costly, and it is important to optimize the sequencing cost by choosing cells which have been amplified uniformly over the whole genome. We have developed a tool Scellector which implements a method to detect amplification quality from shallow coverage data (< 1×) and prioritizes well amplified cells for high coverage sequencing. With the advent of single cell DNA sequencing from companies like Chromium Single Cell CNV profiling solutions (10× Genomics), which uses an isothermal amplification protocol similar to MDA, we believe that our tool can be extended to estimate uniformity of amplification from these platforms. This platform can profile hundred to thousand cells in a single sample to detect copy number variation and provide information on genomic heterogeneity as well as clonal evolution. Not all cells will have uniform amplification and Scellector can be used to detect and remove low quality cells, which will make the downstream analyses of CNV detection more robust. Scelector is an open source tool and source code can be found at https://github.com/abyzovlab/Scellector.

## Conclusion

We have developed a method and its implementation, ‘Scellector’, which uses low coverage whole genome sequencing data for detection of allelic imbalance introduced during whole genome amplification process such as MDA. We have shown our method works very well for detection of ununiformly amplified single cell from low coverage data.

## Methods

### Cell samples origin and genome amplification

Single cell DNA used here for validation of Scellector originated from a human induced pluripotent stem cell line (9230–03#8, Vaccarino Laboratory) differentiated into neurons following an established protocol [23]. Single cells were isolated after 30 days of terminal differentiation by flow cytometry (BD FACS Aria II) in 2.5µL PBS, frozen on dry ice and conserved at − 80 °C before amplification. Amplification using MDA were obtained through Accusomatic service (SingulOmics), which consisted of a custom cold lysis preliminary step followed by amplification with REPLI-g kit (Qiagen) and DNA purification with AMPure XP-beads kit (Beckman Coulter). To be selected for sequencing, amplification samples were selected based on total yield (above 5 µg) and 4-loci PCR test [14].

Bulk DNA sample of induced pluripotent stem cell was used as a reference genome. DNA was purified through DNeasy Blood and Tissue kit (Qiagen) before sequencing at high coverage.

### Sequencing

The low coverage sequencing was conducted at Yale Stem Cell Center Genomics Core facility. The library preparation was done using Nextera XT (DNA library kit, Illumina) and the samples were pooled together to be sequenced on Hiseq4000 (2 × 100 bp) at low coverage per sample (0.1× to 0.4×). For the high coverage sequencing (requested coverage above 30×) of bulk and validated amplified DNA, the library preparation and sequencing (DNBseq) were conducted by the BGI sequencing company (China).

### Data analysis

The bulk sample, shallow and high coverage samples were analyzed using the same pipeline. We started with raw fastq files which were aligned to the GRCh37 human reference genome using BWA mem version 0.7.10 [24], the bam files were then realigned and recalibrated using GATK 3.6. The germline variant calling for the bulk sample was performed using GATK haplotype caller version 3.6(25). The resulting bam files and vcf file were analyzed using Scellector.

## Supplementary information

**Additional file 1.** File containing supplementary figures.

## Data Availability

The sequencing data from this study have been deposited to the NIH NIMH Data Archives under collection number 2424 and submission 31160 ( https://nda.nih.gov/study.html?id=1027).

## Abbreviations

MDA: Multiple displacement amplification
VAF: Variant allele frequency
scDNA-seq: Single cell DNA sequencing
WGA: Whole genome amplification
DOP-PCR: Degenerate oligonucleotide-primed polymerase chain reaction
MALBAC: Multiple annealing and looping-based amplification cycles
MAPD: Median absolute pairwise difference
WGS: Whole genome sequencing
PCR: Polymerase chain reaction
HETs: Heterozygous SNPs
SNPs: Single nucleotide polymorphism
VCF: Variant call format
CNV: Copy number variation
