# Supplementary material for: SCELLECTOR: ranking amplification bias in single cells using shallow sequencing
Source: BMC Bioinformatics. 2020 Nov 12;21:521. doi: 10.1186/s12859-020-03858-y (PMC7663899; doi:10.1186/s12859-020-03858-y)
Supplement: Supplementary file 1 — Additional file 1. File containing supplementary figures. [file 12859_2020_3858_MOESM1_ESM.docx]

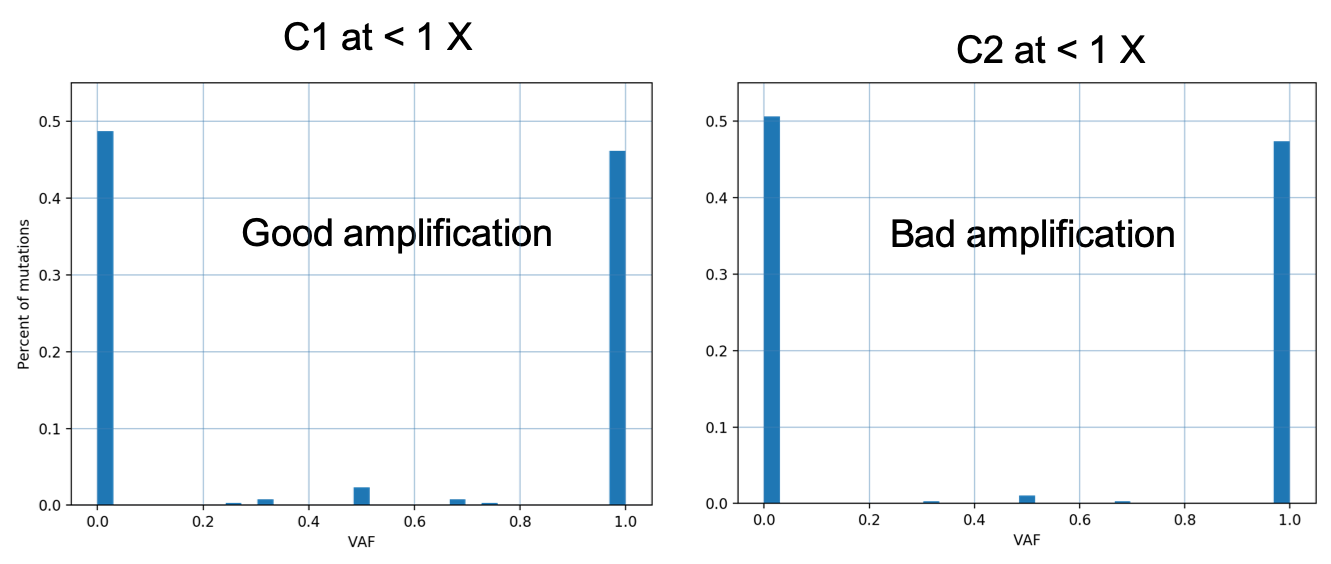


**Figure S1.** Low coverage VAF distribution for cells with good and poor amplification. C1 and C2 are the well amplified and poorly amplified cell respectively from Figure 1. Without using binning and phasing of SNPs, it is impossible to distinguish amplification quality of these cells.


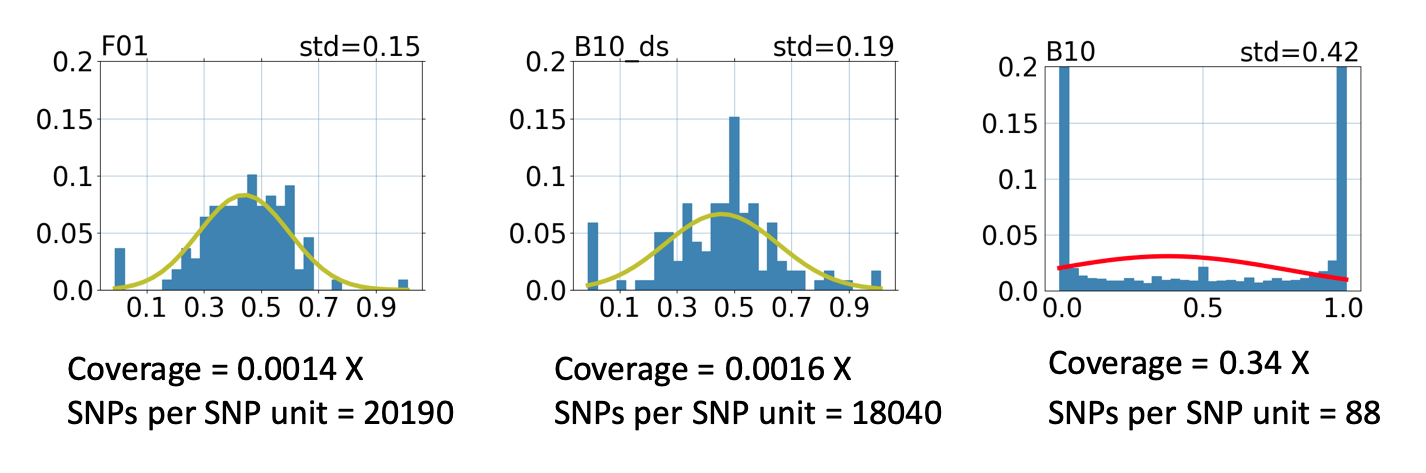


**Figure S2.** Impact of large SNP unit on VAF plots. When the coverage gets lower, the size of the SNP unit gets higher. Cells with very low coverage such as F01, can exhibit properties of uniform amplified cell even though they might be poorly amplified. We took B10 (with bad amplification) and down-sampled it (B10_ds) to match the coverage of F01, to demonstrate that, using very low coverage data is not ideal to determine amplification quality.


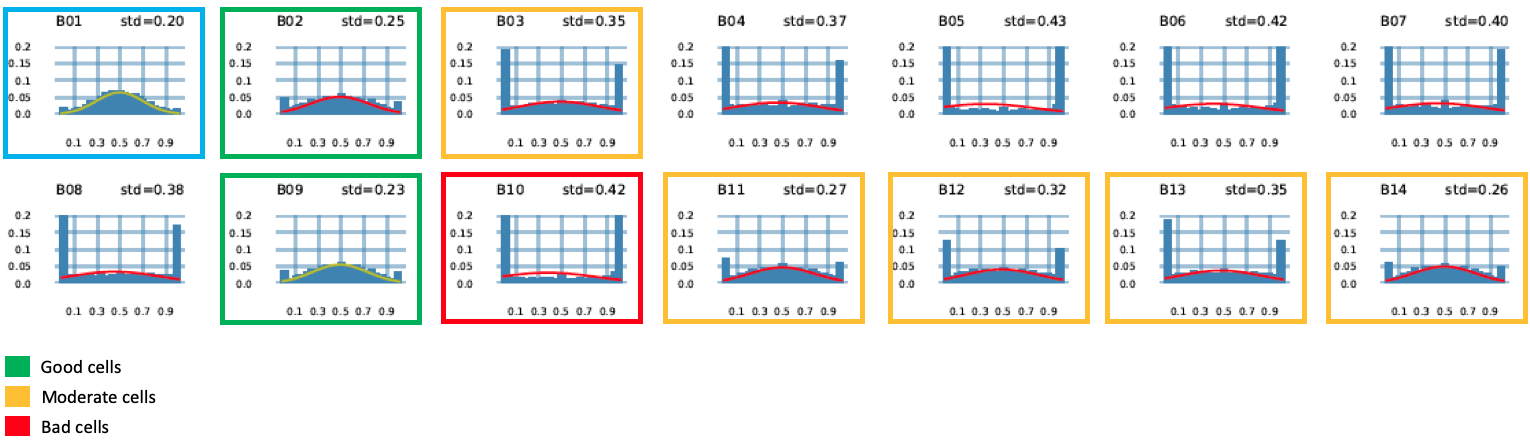


**Figure S3.** Selection of cells for high coverage sequencing. The figure shows VAF distribution of phased SNP units from shallow sequencing for amplified cells. All cells with good (B02 and B09; green squares) and moderate (yellow squares) amplification were selected. One cell with bad amplification (B10; red square) was selected as a negative control. Additionally, we select cell B01 (blue square) with too shallow coverage of 0.11X.


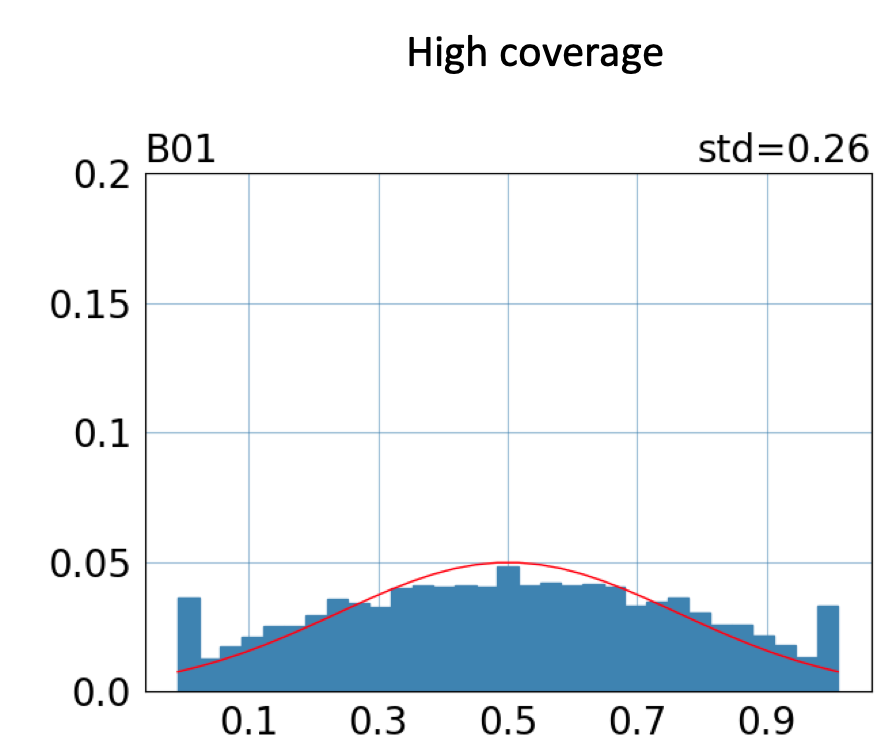


**Figure S4.** Cell B01 has good amplification quality from high coverage.


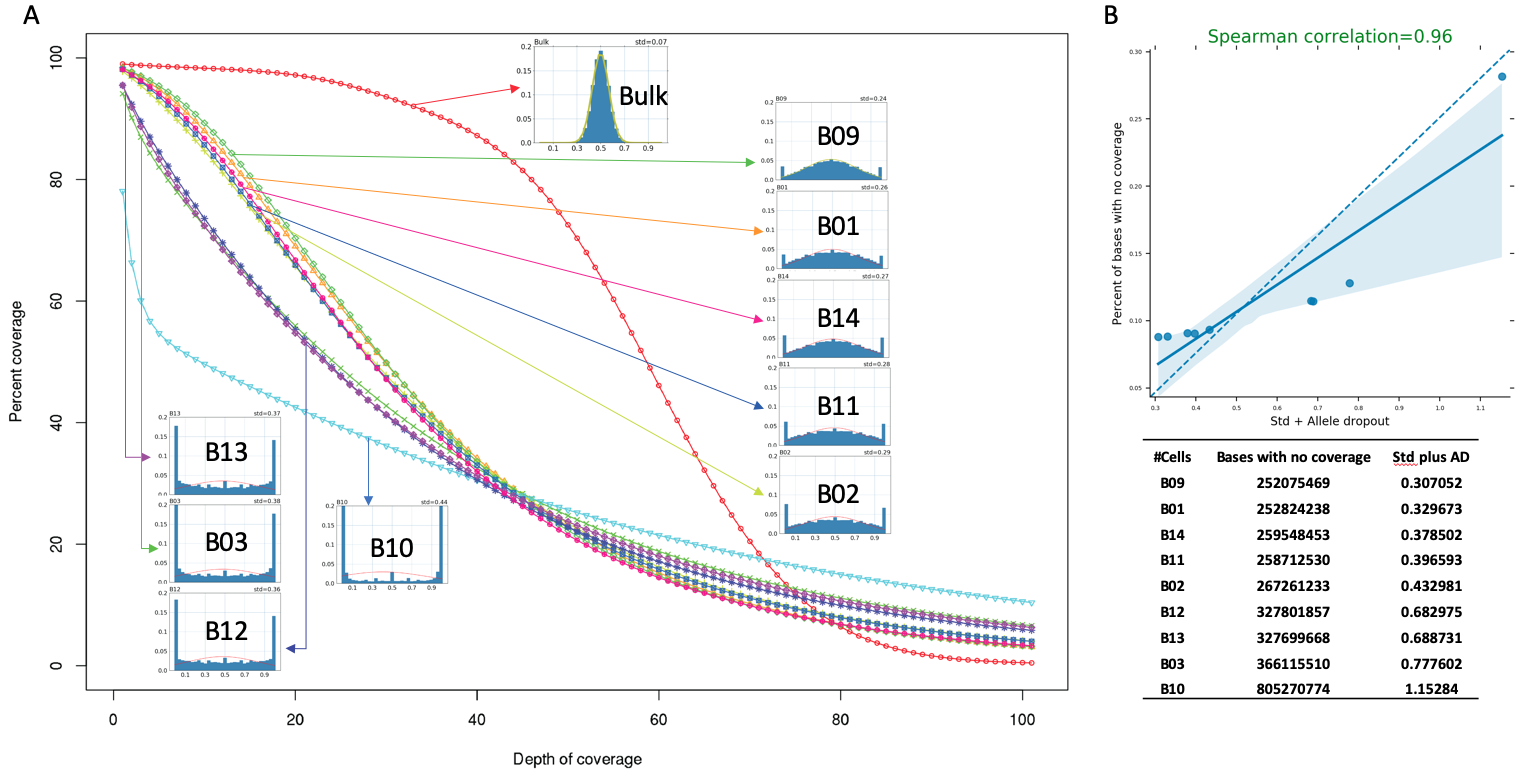


**Figure S5.** Relation between rate of coverage distribution and variant allele frequency plots for each cell with high coverage data. A) The shape of coverage distribution corelates with amplification quality (standard deviation and allelic dropout rate). B) The combined value of standard deviation and allele dropout rate correlates with number of bases not covered in a cell.


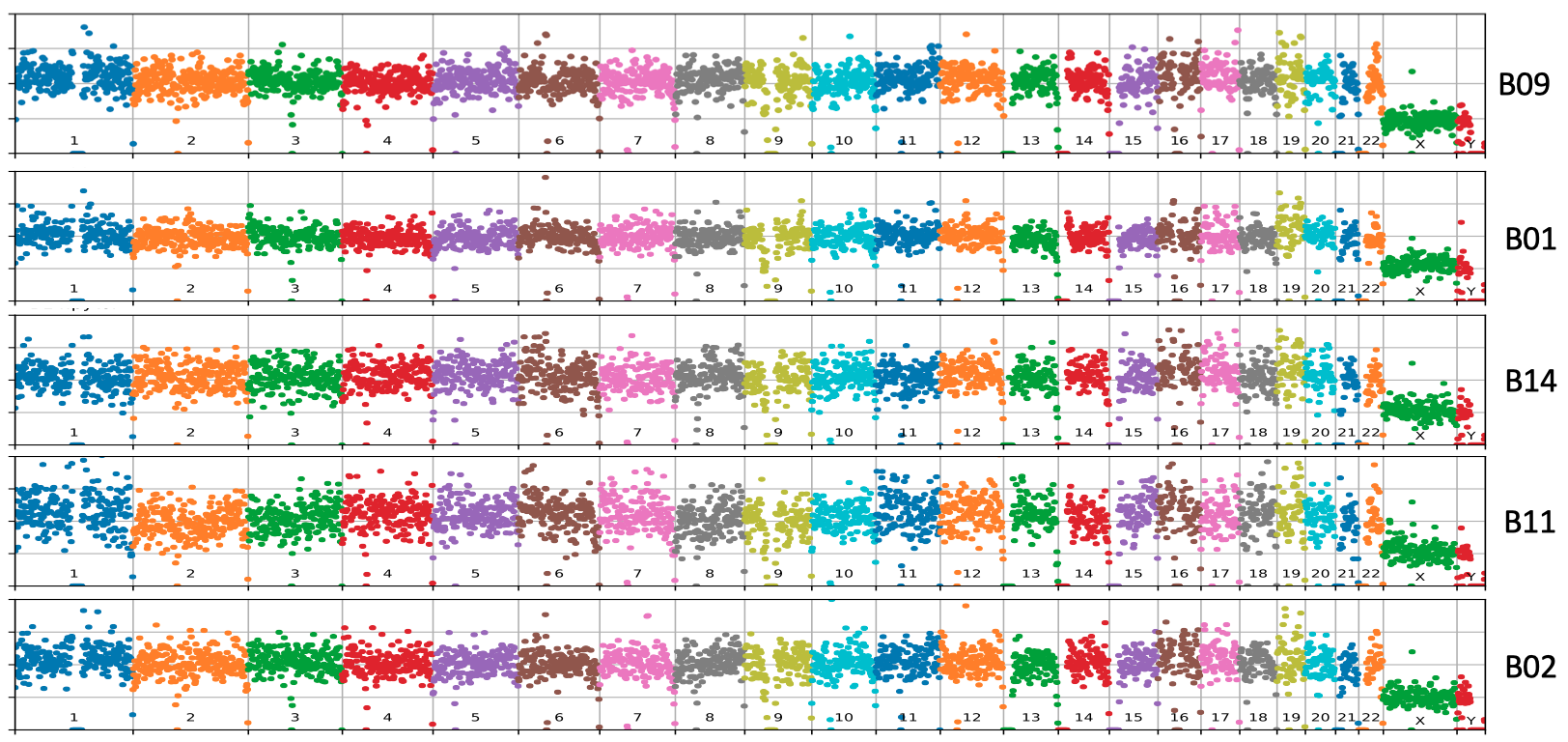


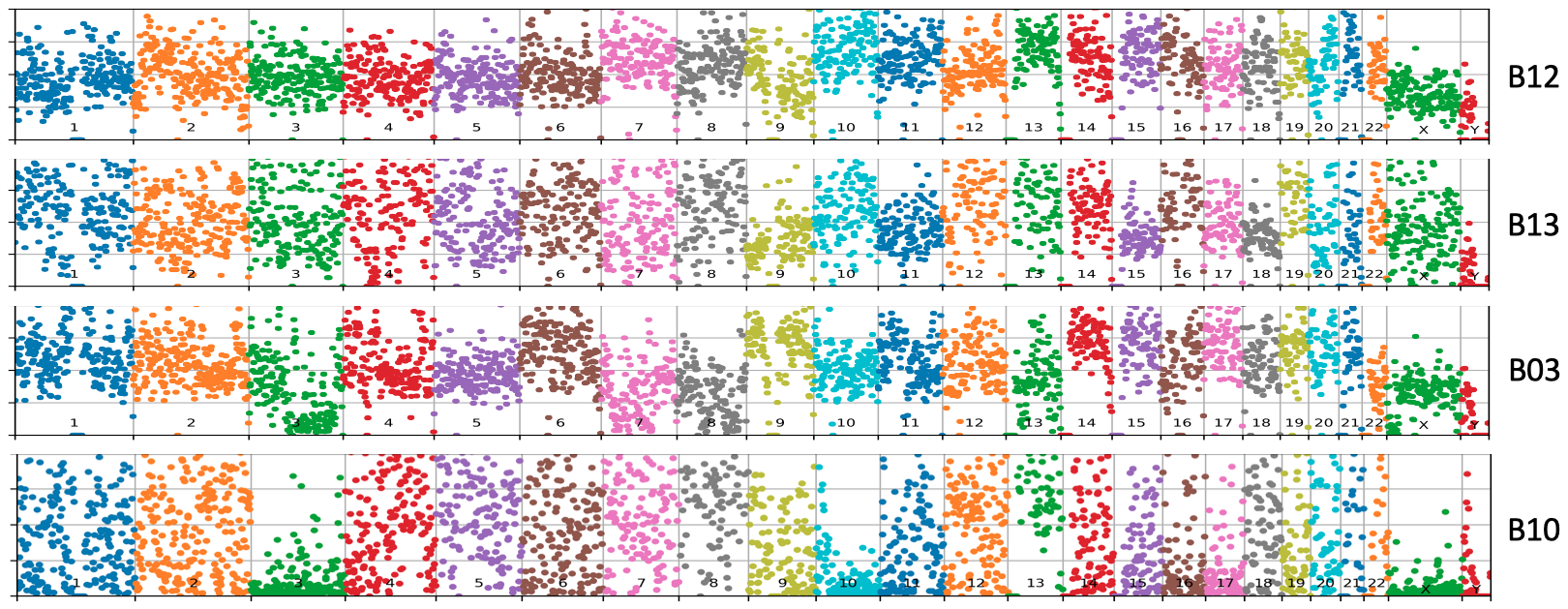


**Figure S6.** Read depth distribution across genome for cells (sorted by standard deviation plus allele dropout score from Scellector). B09 has a tight read depth distribution as compared to unevenly amplified B10. Uneven coverage in B10 will likely affect CNV calling by reducing sensitivity and leading to false positive calls.


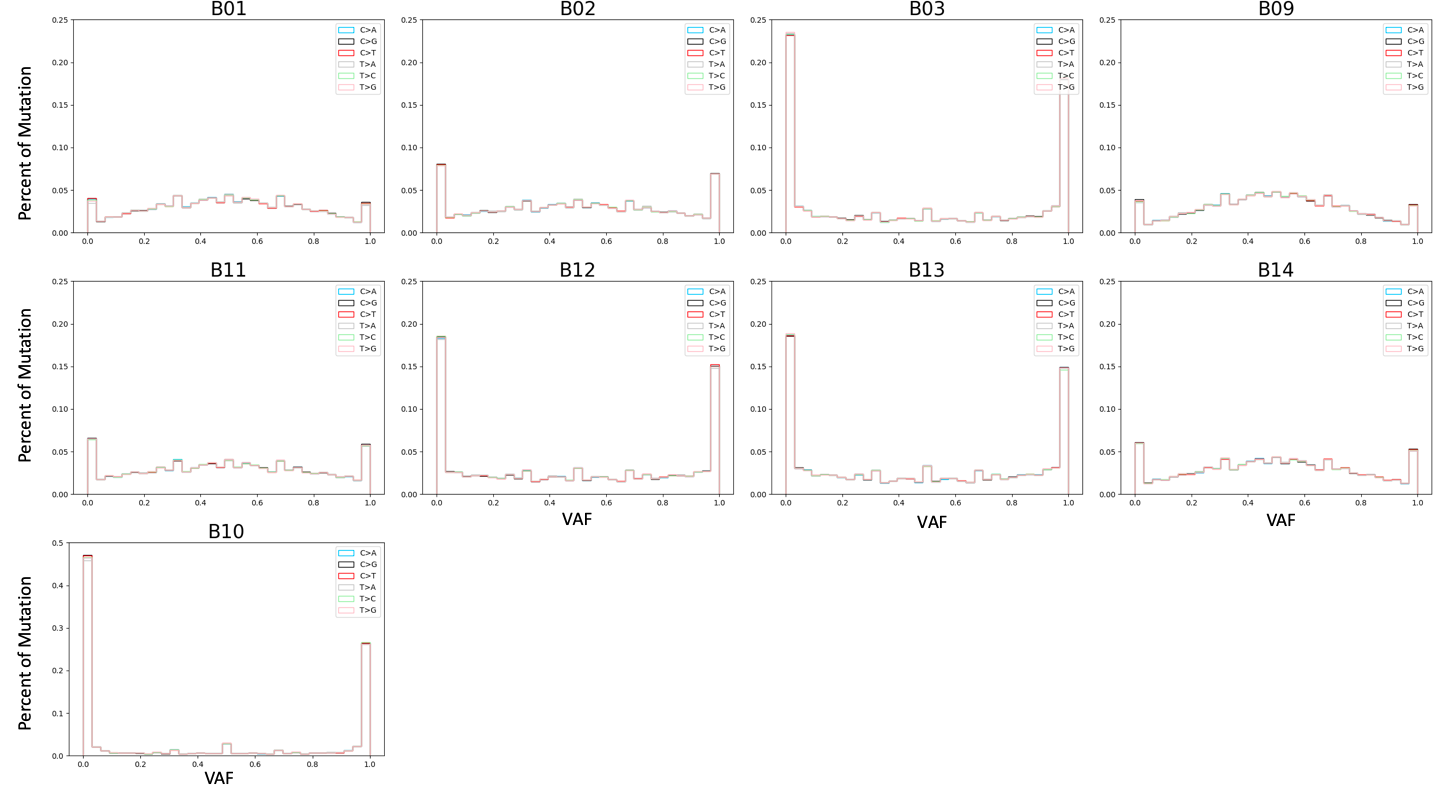


**Figure S7.** Imbalance of amplification is not related to base type. Histogram of variant allele frequency broken down by six mutation types. All mutation types are evenly distributed in the respective variant allele frequency bins.


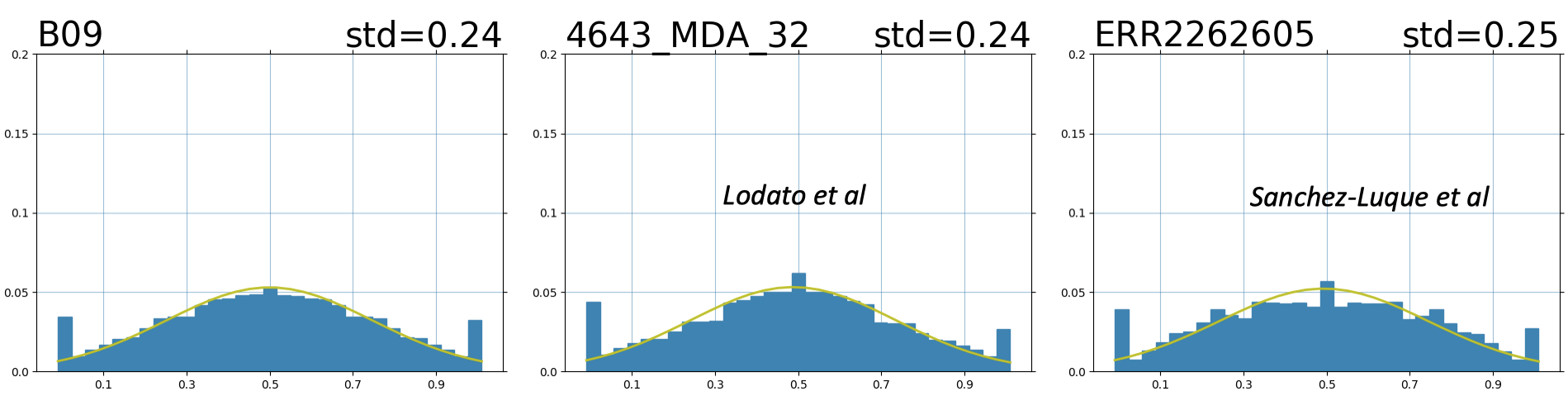


F**igure S8.** VAF plots for cells with best amplification quality across different MDA methods. Comparing our data with data from *Lodato et al, 2015 {PMID:* *26430121}* and *Sanchez-Luq­ue et al, 2019 {PMID:31230816}* we see the standard deviation and allele drop out for well amplified cells being similar.


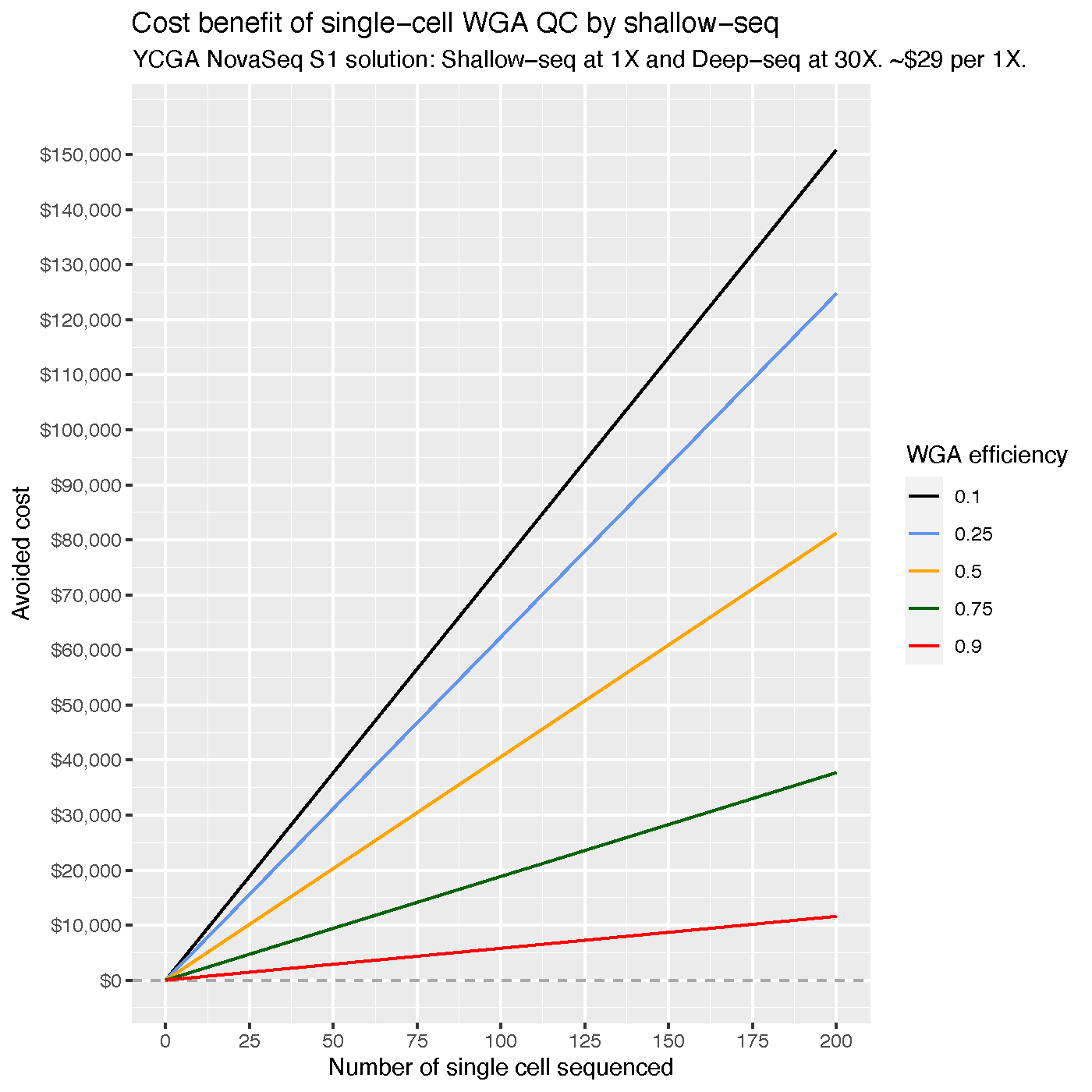


F**igure S9.** Estimated cost saving for amplification QC with shallow sequencing. Here we show the cost savings, if low coverage is being performed at 1X and high coverage at 30X. With WGA efficiency being fraction of well amplified cells, the cost saving increase with decrease in WGA efficiency.


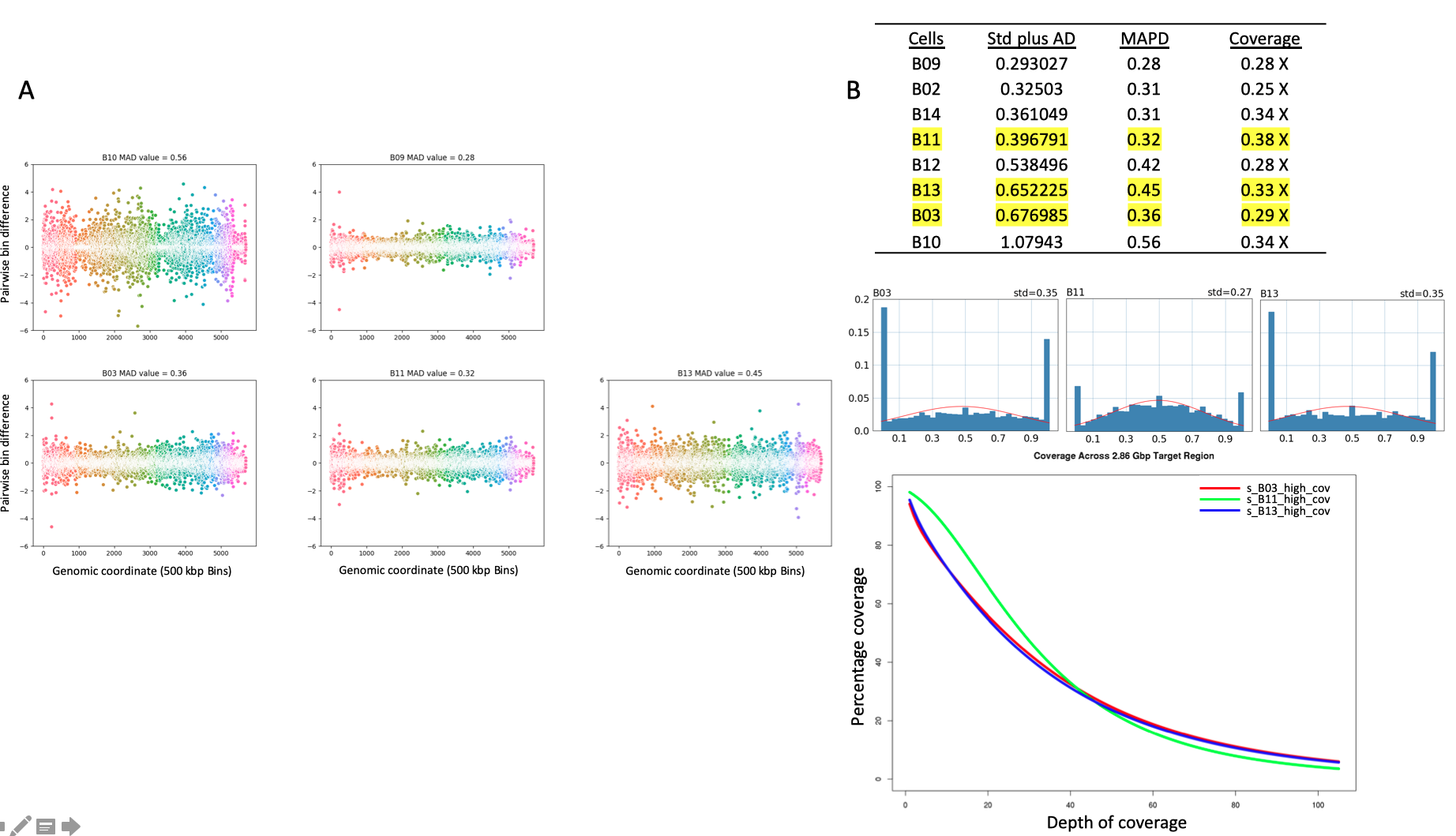


**Figure S10.** Comparing QC approach by Scellector and by MAPD for shallow coverage data. A) MAPD score was able to differentiate between the best amplified cell and the worst amplified cell as we see a wider dispersion of the pairwise bin difference for B10 compared to B09. However, the dispersion of cell B03 and B11 are similar and their MAPD scores are also very similar (the data is colored by chromosome). B) Scellector unambiguously indicates that amplification quality of cell B11 is much better than that for cell B03 and B13 (highlighted scores in the table). The VAF plot also shows an apparent difference between the two cells. Lastly, Scellector reports B03 to be more similar to B13 (MAPD shows B03 to be similar to B11 than B13 in (A)) and the coverage plot clearly shows the coverage profile of B13 is more similar to B03, indicating our method is more sensitive than MAPD.
